# Comparison response patterns on landline and cell phone in a call back survey: effects of demographic characteristics and lag days

**DOI:** 10.13094/SMIF-2019-00019

**Published:** 2019-05-17

**Authors:** Xiaoting Qin, Cathy M. Bailey, Hatice S. Zahran

**Affiliations:** Centers for Disease Control and Prevention, National Center for Environmental Health (NCEH), Asthma and Community Health Branch, Buford, Atlanta, USA

**Keywords:** asthma call-back survey, BRFSS, dual frame surveys, lag day, Response rate

## Abstract

The Asthma Call-back Survey (ACBS) is conducted after the Behavioral Risk Factor Surveillance System (BRFSS) survey by calling BRFSS respondents who reported ever being diagnosed with asthma. To find response patterns and increase ACBS response rates, we first examined whether obtaining consents during the BRFSS survey could increase call back response rates by reducing the refusal and break-off. Then, we assessed how the lag days between BRFSS and ACBS interviews affected response rates. BRFSS cell phone respondents agreed more often to being called back than did landline respondents (75.5 vs. 70.9 percent). However, when respondents were contacted for ACBS, the cell phone response rate was lower than landline response rate (43.4 vs. 47.0 percent), except among males aged 25–34 years, for which the cell phone response rate was 2.1 percent higher than the landline response rate. ACBS response rate for landline and cell phone response were highest if the callback was within 2 days of BRFSS interviews (92.3 vs. 88.8 percent). As lag days increased, the response rate decreased. The cell phone response rate showed a sharper drop; after 2 weeks, the response rate gap between landline and cell phone samples reached 17.3 percent.

## Introduction

The Behavioral Risk Factor Surveillance System (BRFSS) is an ongoing series of health-related telephone surveys conducted by the Centers for Disease Control and Prevention (CDC). Historically, BRFSS had been conducted as a random-digit-dial (RDD) landline survey. The Asthma Call-back Survey (ACBS), referred to as the BRFSS ACBS, is an in-depth asthma survey developed and funded by the Asthma and Community Health Branch (ACHB), the National Center for Environmental Health (NCEH), and CDC. Within two weeks of the BRFSS interview, BRFSS calls BRFSS respondents who reported ever been diagnosed with asthma and consented to participate in the survey.

In 2011, BRFSS started using a dual-frame RDD by including cellular telephones in both the BRFSS main survey and the ACBS in order to produce a more representative sample and higher quality data. The dual-frame RDD was adopted because a growing number of households in the United States are cellular telephone only (CDC 2012a, 2012b). In fact, data from the National Center for Health Statistics indicate that 54.9% of American homes did not have a landline telephone but did have at least one wireless telephone — an increase of 2.4 percentage points for wireless telephones-only households since the first half of 2017 (Blumberg and Luke 2018). The use of a dual-frame RDD survey that includes landline and cellular telephones has improved the BRFSS data’s validity, quality, and representativeness (CDC 2017).

Studies published after the implementation of the dual-frame RDD in telephone surveys have shown that landline and cell phone sample frames differed in response patterns. A comparison of the BRFSS landline and cell phone response measures showed that landline surveys have higher response rates than cell phone surveys, but cell phone respondents show higher cooperation rates (Qayad et al. 2013). A study based on the National Flu Survey showed that maximized respondent contact and completed interviews might not be the most cost-effective for cell phone surveys (Reimer et al. 2012). Another study found that landline and cell phone samples differ substantially about best time to call, and that contact rates decline after repeated dialing (Montgomery 2011).

In addition, research shows that people who have only cellular telephones have a different demographic profile and response pattern in a survey, compared to people who have landline telephones. People who have only cellular telephones tend to be younger and unmarried, rent instead of own a home, and are predominately Hispanic (Blumberg and Luke 2016). Furthermore, more than seven in ten adults aged 25–34 live in households with wireless-only service, compared to six in ten adults aged 18–24. The percentage of adults living with only wireless telephones decreased as age increased beyond 35 years. For example, less than one quarter (23.5 percent) of adults over age 65 live in wireless-only homes (Blumberg and Luke 2016). Keeter et al. (2017) showed that increasing cellphone samples has improved representation of young adults and Hispanics. Kennedy et al. (2016) indicated that subgroup differences are largely confined to estimates for ages 65 and plus between landline and cell phone, and cell phone penetration rates are not uniform across all segments of the population. These studies show that having a dual-frame RDD survey gives the opportunity to expand the coverage to people of all ages and improves survey response rate. However, most of these studies were based on single-phase telephone survey data, such as the BRFSS, and not on two-phase telephone survey data, such as aCbS. It is conducted among BRFSS respondents who reported ever being diagnosed with asthma (CDC 2015). To the best of our knowledge, for a callback survey, whether having a dual-frame RDD survey can also improve the coverage to people of all ages or minority groups remains unknown. Assessing the differences in response pattern among demographic groups will identify subgroups with low response rates. This may help us to focus on those groups to improve their response rate and produce better, more representative data.

When conducting a call back survey, two key factors could play a role in response rate: consents and lag days between BRFSS and ACBS. The number of BRFSS respondents with asthma who agreed to participate in ACBS when asked at the end of the BRFSS interview (consent rate) will affect the number of eligible ACBS respondents, which affects the response rate as described in the Methods. Information on demographic characteristics of those consented to participate in the ACBS and the best waiting period between the two interviews (lag days) could be used in developing more effective calling strategies for improving ACBS landline and cell phone response rates.

This study aims to answer the following questions: 1) how do demographic subgroups differ in obtaining consent for participation in the call back survey, and 2) how do the lag days between BRFSS and ACBS affect response rates. For both landline and cell phone samples, we assessed the effects of lag days on the response rate and computed the rate of eligible BRFSS respondents who agreed to be called back, ACBS response, and refusal/break-off rates by demographic characteristics.

## Methods

We analyzed BRFSS and ACBS data from 2012 and 2013 to obtain stable estimates. For the analysis, data from 29 states that participated in both the landline and the cell phone surveys were included; the data from states that participated only in the landline phone survey were excluded.

States conducted BRFSS and ACBS separately, then submitted data in a standard format to CDC. States conducted ACBS using the same procedures as specified in the BRFSS calling guidelines (CDC 2013a). We managed the cellular telephone sample in a manner similar to the landline telephone sample. For landline and cellular telephone surveys, ACBS used a single set of disposition codes adapted from standardized American Association of Public Opinion Research (AAPOR) disposition codes for telephone surveys (AAPOR 2016). A few disposition codes apply only to landline telephone or cellular telephone sample numbers. The disposition code and sample categories can be found on the ACBS website (CDC 2013b).

The median state prevalence rate for BRFSS 2012 and 2013 respondents who reported ever being diagnosed with asthma was 8.9 percent and 9.0 percent, respectively (CDC 2016). That meant in this study, 63,357 BRFSS respondents were eligible for ACBS.

ACBS eligible respondents were asked a recruiting consent question at the end of the BRFSS interview. We used the responses to that question to compute the rate of eligible persons who agreed to participate in ACBS. We calculated response rates and cooperation rates using AAPOR formulas (AAPOR response rate 4 [RR4] and cooperation rate 2 [COOP2], respectively) (AAPOR 2016). We also computed the refusal/break-off rate for eligible respondents who refused to be called back in BRFSS or did not answer ACBS questions. We calculated response rates and cooperation rates by sex (male and female), age (18–24, 25–34, 35–44, 45–54, 55–64, and 65 years and over), and race/ethnicity (non-Hispanic whites, blacks, other, multiracial, and Hispanics). Because the cooperation and response rates showed similar patterns, we report only response rates in this paper. We also tested the rate differences between the landline and cell phone samples using the Pearson chi-square test.

We used the following formulas to calculate the rates:

Agree to be called back rate = (Yes to be called back / BRFSS asthma eligible respondents who were asked whether to participate for ACBS)*100

Eligibility factor = Contacted eligible at ACBS / (Contacted eligible at ACBS + Ineligible for Asthma Call-back at BRFSS + Ineligible at ACBS + No ACBS attempt)

Response rate = (Completed interviews / (Contacted eligible at ACBS + (Eligibility factor * Not interviewed for technical problems)))* 100

Cooperation rate = (Completed interviews^¥^ / (Contacted eligible at ACBS + Terminations and refusals at ACBS))*100

Refusal rate = ((Terminations and refusals at BRFSS + Terminations and refusals at ACBS) / (Contacted eligible at ACBS + (Eligibility factor * Not interviewed for technical problems))) * 100

In addition, we assessed the effect of lag days on ACBS completion by computing the response rates by lag days, then comparing the differences between the landline and cell phone samples. The calling protocol recommended that eligible respondents be interviewed within 2 weeks of the BRFSS interview. The states could deviate from the protocol and only about 25% of the sample was interviewed within 2 weeks of the BRFSS interview. This occurs often when the interviewers for ACBS and BRFSS are different. While transferring ACBS eligible respondents from BRFSS into the ACBS sample pool, the ACBS interviewer may not be able to get the calling number within 2 weeks.

Lag day are the number of days between BRFSS interview completed date and ACBS interview date when the final disposition code was assigned. For the analysis, lag days were categorized as 0–2 days, 3–4 days, 5–7 days, 8–14 days, 15–21 days, and more than 21 days. We used a Cochran-Armitage trend test (Liu 2008) to determine whether the lag days had progressive effects on response rate. Commonly used in categorical data analysis, the Cochran-Armitage trend test is appropriate to detect an association between a variable with two categories (landline vs. cell phone) and an ordinal variable with multiple categories (lag days with multiple categories). The null hypothesis is the hypothesis of no trend, which means that the binomial proportion is the same for all levels of the explanatory variable.

## Results

Table 1 shows the progression from 2012 and 2013 BRFSS eligible respondents to ACBS sample. A total of 65,371 BRFSS adult respondents were eligible for ACBS. Among those, 37,824 were contacted and interviewed. About 42 percent of eligible respondents did not participate in ACBS, for three primary reasons: were not asked about being called back, refused to be called back, and were not contacted after consenting to the Call-back survey (Table 1).

### Overall ACBS Landline and Cell Phone Response Patterns

**Figure 1:**
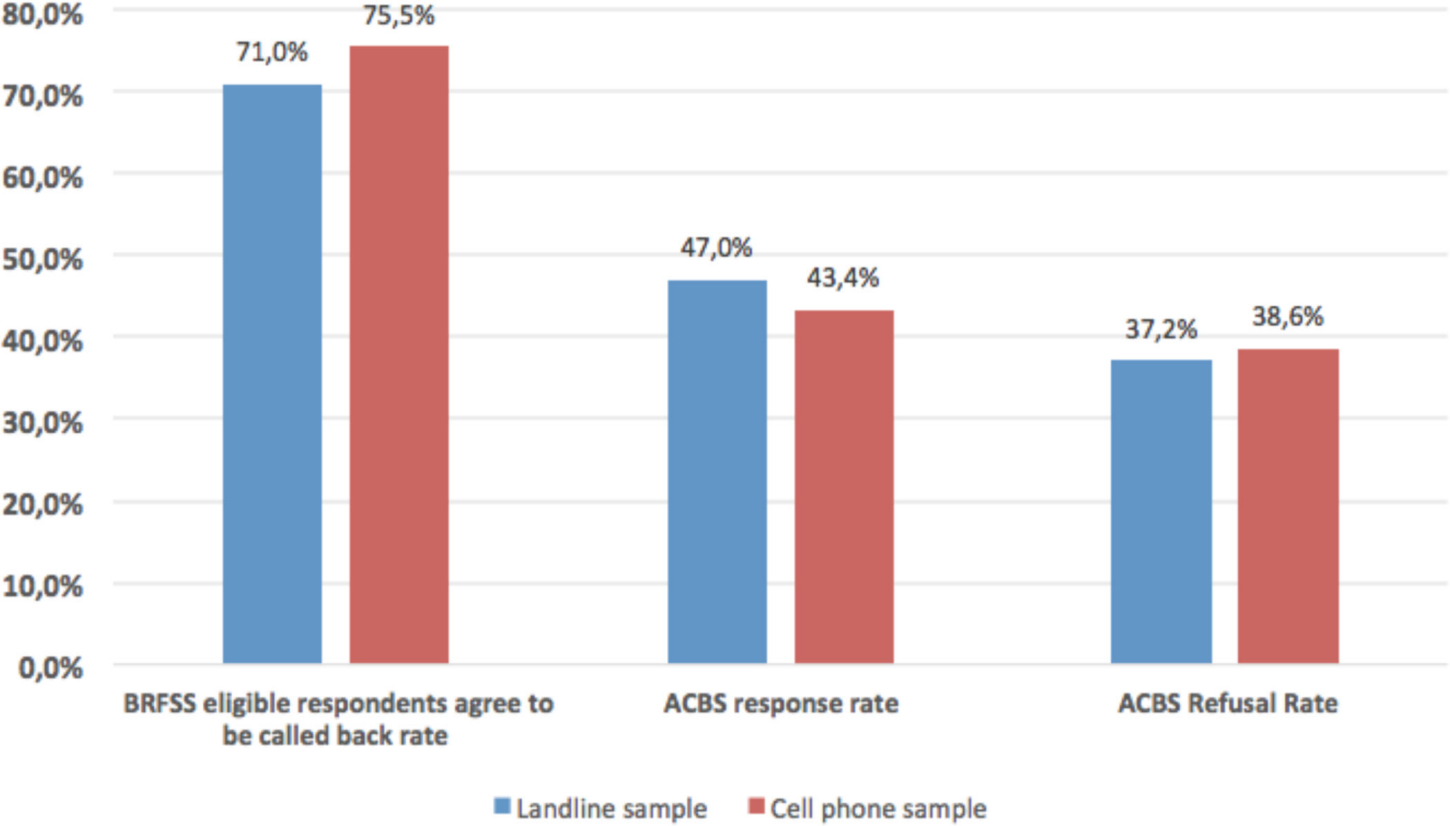
Rate difference between landline and cell phone

Overall, in BRFSS interviews, cell phone respondents were more likely to agree to participate in ACBS than were landline respondents (75.5 percent vs. 71.0, *p*<0.0001) (Figure 1).

The ACBS cell phone sample had a lower response rate (43.4 percent vs. 47.0 percent, *p*<0.0001), and a higher refusal rate (38.6 percent vs. 37.2 percent, *p*=0.0741) than did the landline sample (Figure 1).

### Agreed-to-Be-Called-Back and ACBS Response Rates by Demographics

**Figure 2:**
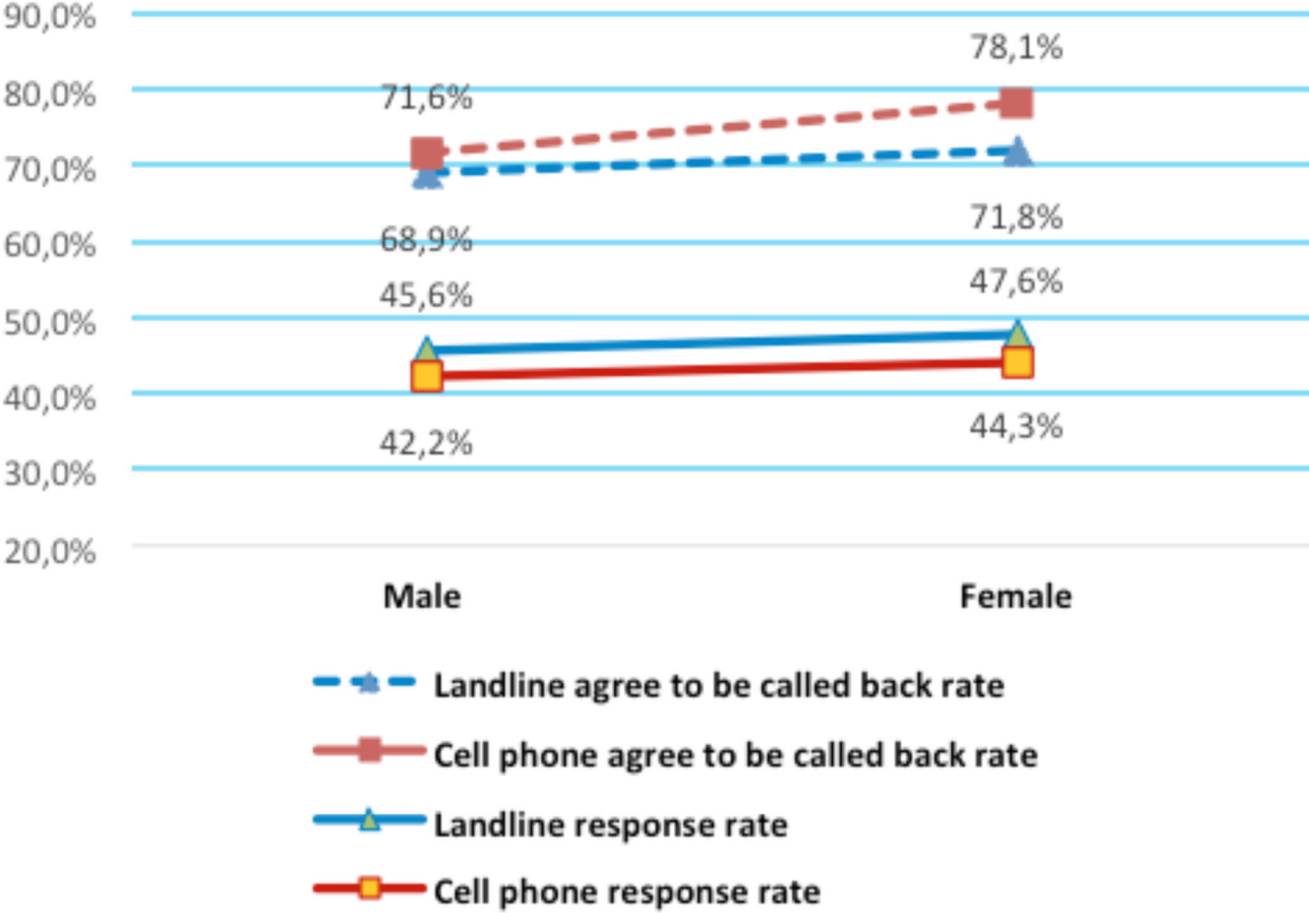
Rate comparisions by sex

As seen in Figure 2, for both sexes, the percentages of eligible participants who agreed to be called back were higher for the cell phone samples than for landline samples (female: 78.1 percent vs. 71.8 percent, *p*<0.0001; male: 71.6 percent vs. 68.9 percent, *p*=0.0002). However, the cell phone samples had lower response rates compared with landline samples (female: 44.3 percent vs. 47.6 percent, *p*<0.0001; male: 42.2 percent vs. 45.6 percent, *p*<0.0001). Compared separately, females had higher agreed-to-be-called-back rates (landline: 78.1 percent vs. 71.6 percent, *p*<0.0001; cell phone: 71.8 percent vs. 68.9 percent, *p*=0.0002) and higher response rates (landline: 47.6 percent vs. 45.6 percent, *p*<0.0001; cell phone: 44.3 percent vs. 42.2 percent, *p*<0.0001) than did males.

**Figure 3:**
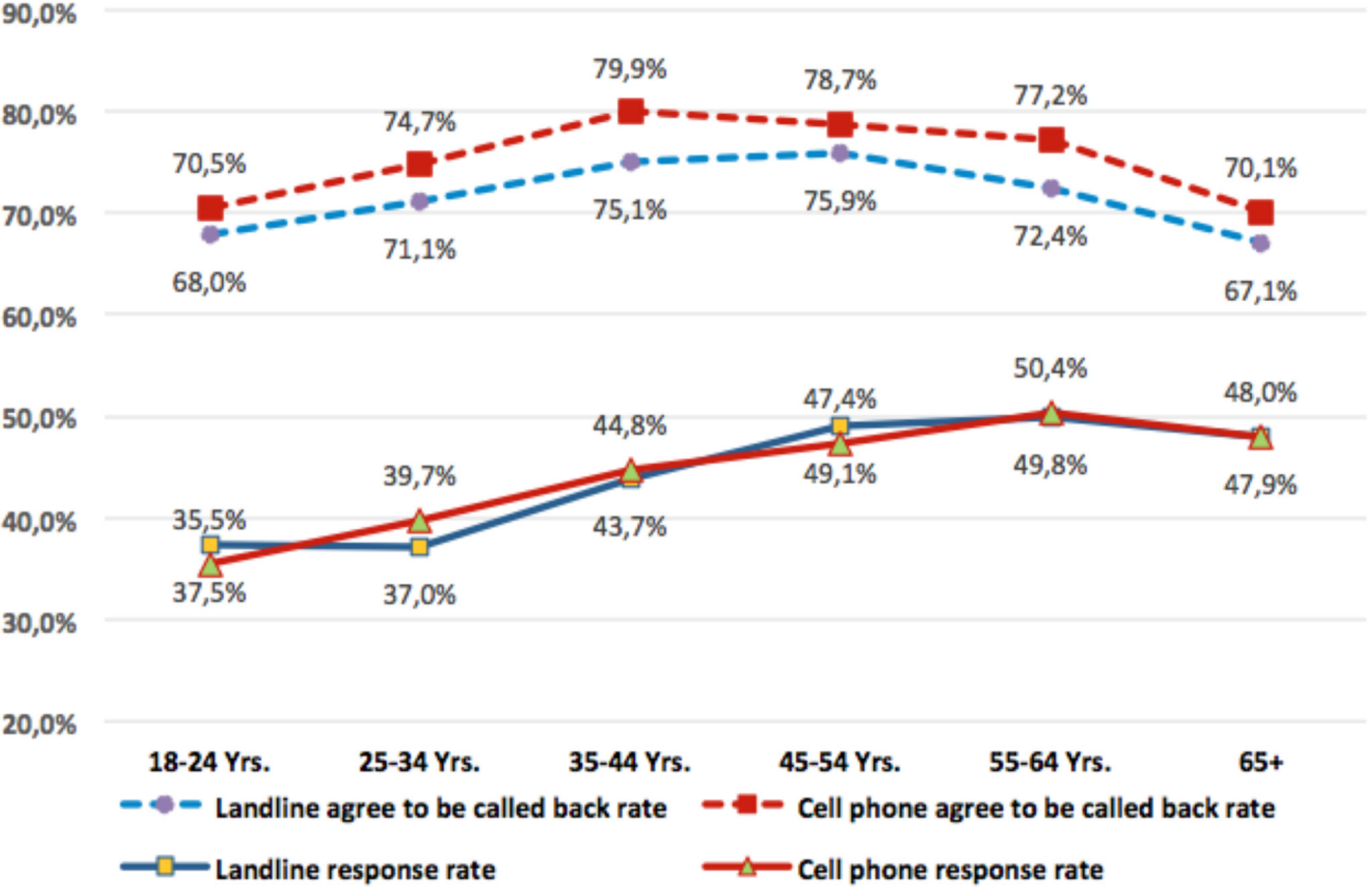
Rate comparisions by age

Figure 3 shows the percentages of BRFSS respondents who agreed to be called back and the response rates by age groups (18–24, 25–34, 35–44, 45–54, 55–64, and 65 or more years). For all age groups, those participating by cell phone had a higher agreed-to-be-called-back rate than did the landline sample (*p*<0.0001). For the ACBS response rate, no significant differences were found between the landline and cell phone samples, except for the 25–34 years age group cell phone sample, which had a higher response rate than did the landline sample for that age group (39.7 percent vs. 37.0 percent, *p*<0.0001).

**Figure 4:**
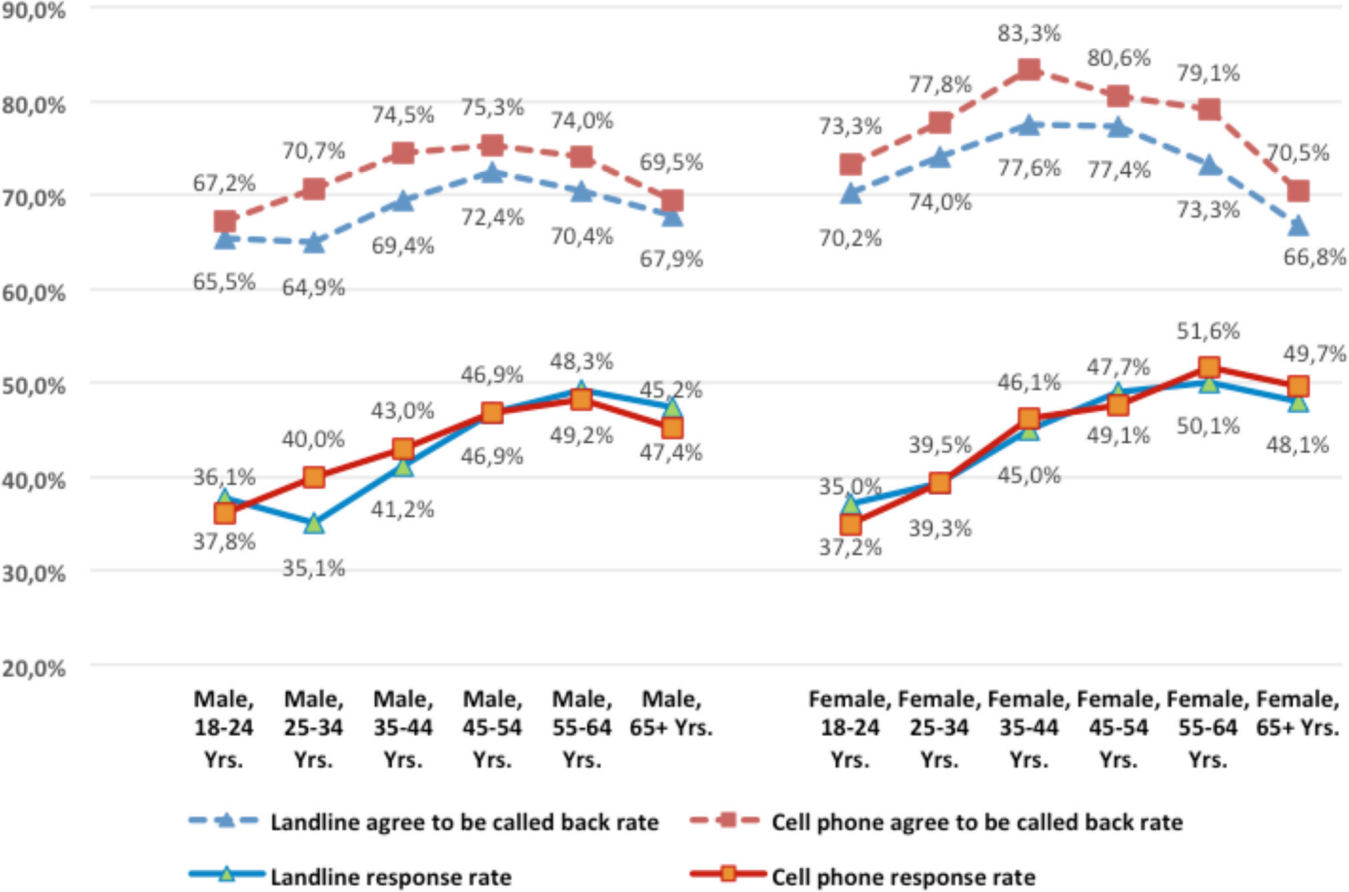
Rate comparisions by age and sex

We also compared response rates by age (18–24, 25–34, 35–44, 45–54, 55–64, and 65 or more years) for both sexes. For all age-by-sex groups, the rates for cell phone BRFSS respondents who agreed to be called back were significantly higher than the rates for landline respondents (*p*<0.0001) (Figure 4). For the 18–24 age group, both male and female, the cell phone samples have a significant higher response rate (*p*<0.0001). For the 25–34 age groups, the patterns for males and females differed. Among males, the cell phone sample had a significantly higher response rate than did the landline sample (40.0 percent vs. 35.1 percent, p<0.0001). For females, the cell phone and landline response rates were similar (39.5 percent vs. 39.3 percent), and no significant difference was found (p=0.3817).

**Figure 5:**
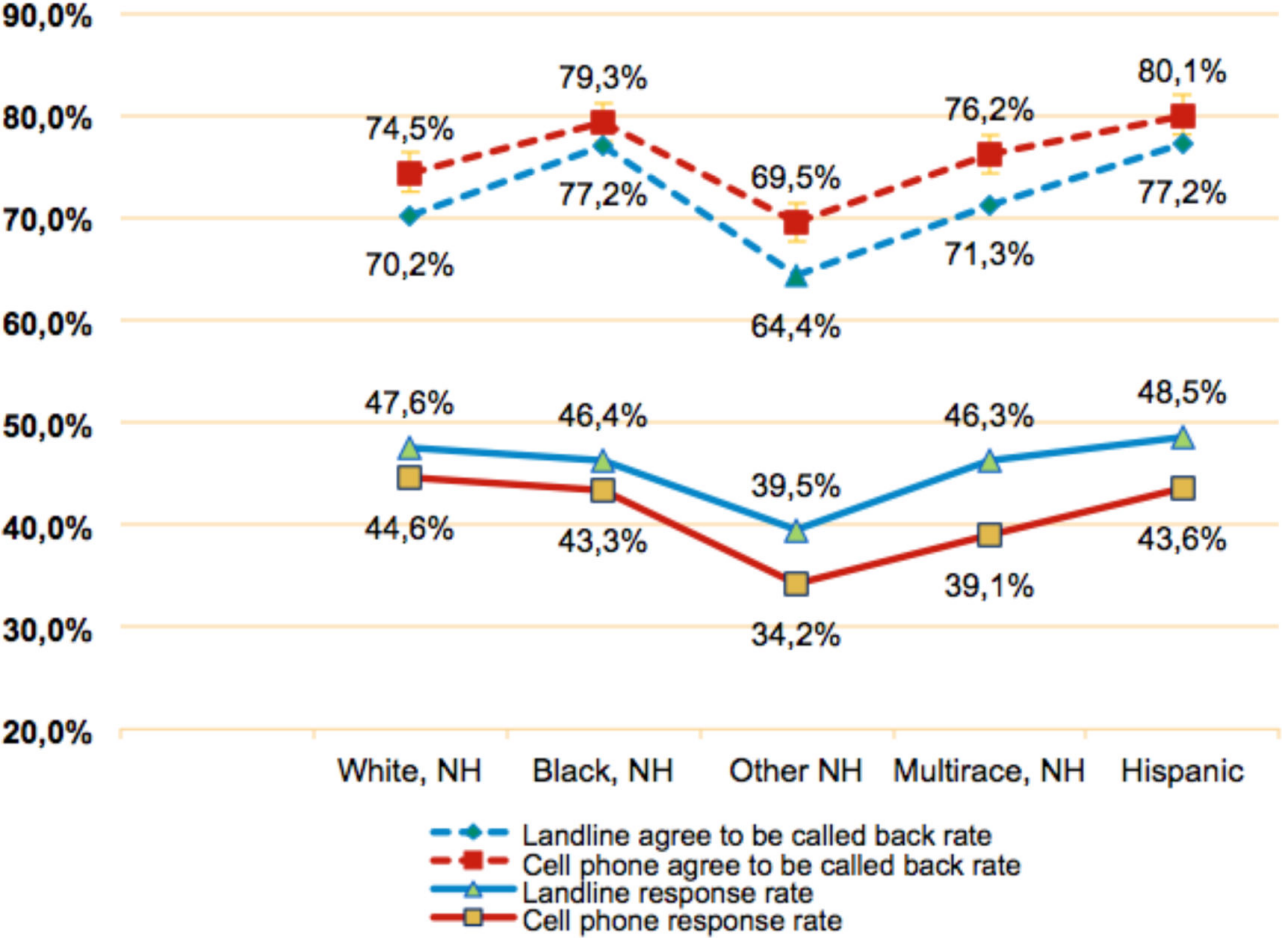
Rate comparisions by race/ethnicity NH = non-Hispanic

We compared the rates of BRFSS respondents who agreed to be called back and response rates for five racial/ethnic groups: non-Hispanic whites, blacks, other, multiracial, and Hispanics. For each group, those in the cell phone sample were more likely to agree to be called back than were the landline sample (Figure 5). Significance was *p*<0.0001 for most of the groups. For the non-Hispanic black (*p*=0.1371) and Hispanic groups (*p*=0.0109), the difference was not significant at *p*<0.0001. The cell phone samples had significantly lower response rates than did landline samples for all five racial/ethnic groups (*p*<0.0001).

### Effects of Lag Days on ACBS Response Rates

**Figure 6:**
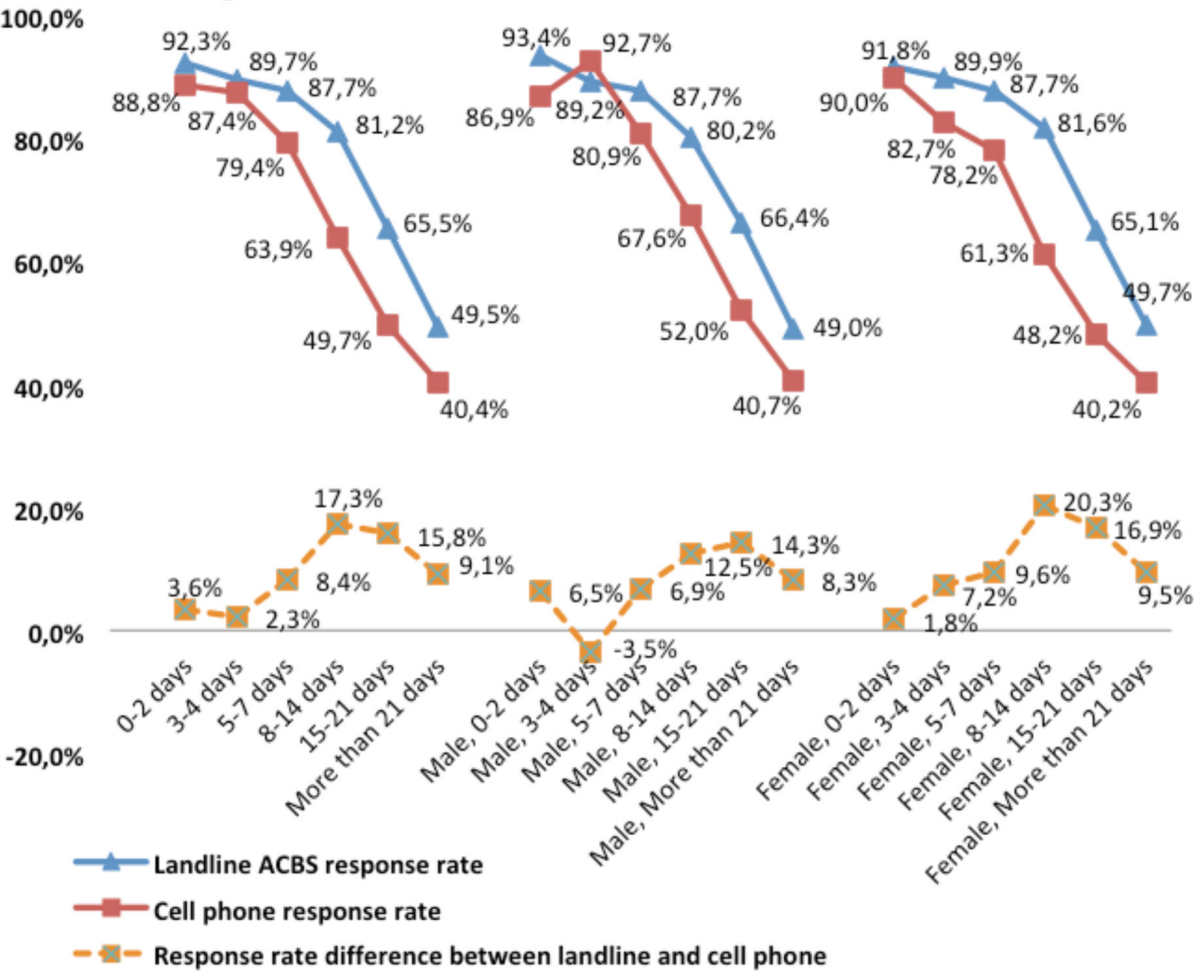
Effect of lag days on ACBS response rate

As seen in Figure 6, the response rates for ACBS landline and cell phone respondents were highest if the callback was within 2 days of the BRFSS interview (92.3 percent and 88.8 percent, respectively). As lag days increased, ACBS response rates decreased, especially for cell phone responses. The gap was greatest (17.3 percent) in the 8–14 days period. A Cochran-Armitage trend test indicated a significant decreasing trend in the response rates (*p*<0.0001).

We also grouped the samples by sex to access the influence of lag days on response rate and found slight differences (Figure 6). The cell phone response rate was higher than the landline response rate for males if the ACBS calls were made between 3–4 days (92.7 percent vs. 89.2 percent, *p*<0.0001). No such pattern was found for the female group. The response rates for females were always lower for the cell phone sample than for the landline sample, no matter the number of lag days. For females, the response rate difference between landline and cell phone samples was greatest (20.3 percent) at 8–14 days. For males, the widest gap (14.3 percent) was at 15–21 days. The effect of lag days was greater and more time sensitive among females than among males.

## Discussion

Our analysis of 2013 data indicates that the difference in response rates between ACBS landline and cell phone samples (4.0 percent) was smaller than the difference in the corresponding BRFSS response rates (11.8 percent) (CDC 2013c). The smaller difference between landline and cell phone response rates in the ACBS is mainly because the cell phone response rate was about 5 percent higher than the corresponding BRFSS rates, and the landline response rate was about 2 percent lower than the corresponding BRFSS rates. We believe that the increase in ACBS cell phone response rate happened because BRFSS respondents were called directly without prior consent, but ACBS respondents were asked consent to participate. Obtaining consent could increase the eligibility factor, which leads to higher response rate. In addition, being aware of a future survey may reduce refusal and break-off, also increasing response rate. However, our findings indicate that while obtaining consent might have improved the ACBS cell phone response rate, it did not have much effect on increasing landline response rate. Further studies must assess the effects of obtaining consent for survey participation on ACBS cell phone and landline response rates.

The RDD cell phone frame improves coverage of many demographic groups (young adults, males, minorities, etc.) within the U.S. general population who have become hard to survey through the landline RDD frame (AAPOR 2010, AAPOR 2017). ACBS cell phone samples had a lower response rate than did landline samples (43.4 percent vs. 47.0 percent, *p*<0.0001). The one exception is males aged 25–34. That group had a significantly higher response rate among the cell phone sample than among the landline sample (39.7 percent vs. 37.0 percent, *p*<0.0001). This finding demonstrated that adding a cell phone sample into the RDD survey is important to reach the hard-to-reach survey group of young male adults. In addition, the result indicated that response rate for the cell phone samples was significantly lower than landline samples for all five racial/ethnic groups. To gain better representation for minority groups, it is important to explore other strategies to improve the ACBS cell phone response rate among minority groups, given that cell phone ownership was higher among minority groups (Blumberg and Luke 2016).

The percentage of eligible BRFSS cell phone respondents who agreed to be called back to participate in ACBS was greater than the percentage of landline respondents (around 4 percent more). However, when conducting the Call-back Survey, more cell phone respondents refused to participate or lost contact. This led to a lower response rate for the cell phone samples compared with the landline samples (around 4 percent less). The lower response rate, which might have resulted from using the same call-back procedures as for the landline sample, could be improved with proven callback strategies:

- Ensuring that interviewers’ phone numbers show up as “Asthma Call-back Survey” in caller identification.
- Increasing the required minimum number of attempted calls.
- Sending a short notification text message before calling.
- Setting up a call-back time for the follow-up call (Dal Grande 2016; Vicente 2017).
- Future research is warranted to assess the effectiveness of these strategies in improving Asthma Call-back Survey cell phone sample response rates.

Through this study, we also found that as lag days increased, ACBS response rate decreased. The cell phone response rate showed a sharper drop relative to the landline sample. After 2 weeks, the response rate gap between the landline and cell phone samples reached 17.3 percent. The important lesson we learned from this finding was that a call-back within 2 days instead of 2 weeks would improve landline and cell phone response rates. However, reducing the lag days for the ACBS cell phone sample is more critical than for the landline sample; the ACBS cell phone response rate showed a sharper drop relative to the landline sample.

As in this study, response pattern differences between ACBS cell phone and landline samples show that the response rate might be improved by modifying the protocol and making sure that eligible BRFSS respondents are contacted within 2 days. These findings could also be used in the future to develop efficient landline and cell phone sample calling strategies to maximize response rates not only for ACBS but also other call-back surveys, such as the BRFSS Zika virus and flu vaccine call-back surveys.

## Figures and Tables

**Table 1:** Comparison of ACBS-eligible BRFSS respondents to ACBS samples

Sample categories			Landline sample count	Cell phone sample count	Landline and cell phone sample –total count
Reported ever being diagnosed with asthma during the BRFSS survey			46,168	19,203	65,371
BRFSS respondents were not asked if they consent to participate in ACBS, ineligible for ACBS			4,533	3,928	8,461
BRFSS respondents were asked if they consent to participate in ACBS			41,635	15,275	56,910
	Refused to be called		12,091	3,747	15,838
	Agreed to be called		29,544	11,528	41,072
		Not contacted	1,940	1,308	3,248
		Contacted for ACBS Interview	27,604	10,220	37,824
